# Ultrafast Intrinsic Photoresponse and Direct Evidence of Sub-gap States in Liquid Phase Exfoliated MoS_2_Thin Films

**DOI:** 10.1038/srep11272

**Published:** 2015-07-15

**Authors:** Sujoy Ghosh, Andrew Winchester, Baleeswaraiah Muchharla, Milinda Wasala, Simin Feng, Ana Laura Elias, M. Bala Murali Krishna, Takaaki Harada, Catherine Chin, Keshav Dani, Swastik Kar, Mauricio Terrones, Saikat Talapatra

**Affiliations:** 1 Department of Physics, Southern Illinois University Carbondale, Carbondale-IL 62901; 2 Department of Physics and Center for 2-Dimensional and Layered Materials, The Pennsylvania State University, University Park, PA 16802; 3 Femtosecond Spectroscopy Unit, Okinawa Inst. of Science & Technology, Graduate University, Onna-son, Okinawa, 904 -0495 Japan; 4 Department of Physics and George J. Kostas Research Institute, Northeastern University, Boston, USA; 5 Department of Chemistry and Department of Materials Science and Engineering, The Pennsylvania State University, University Park, PA 16802

## Abstract

2-Dimensional structures with swift optical response have several technological advantages, for example they could be used as components of ultrafast light modulators, photo-detectors, and optical switches. Here we report on the fast photo switching behavior of thin films of liquid phase exfoliated MoS_2_, when excited with a continuous laser of λ = 658 nm (E = 1.88 eV), over a broad range of laser power. Transient photo-conductivity measurements, using an optical pump and THz probe (OPTP), reveal that photo carrier decay follows a bi-exponential time dependence, with decay times of the order of picoseconds, indicating that the photo carrier recombination occurs via trap states. The nature of variation of photocurrent with temperature confirms that the trap states are continuously distributed within the mobility gap in these thin film of MoS_2_, and play a vital role in influencing the overall photo response. Our findings provide a fundamental understanding of the photo-physics associated with optically active 2D materials and are crucial for developing advanced optoelectronic devices.

Single- and few-layers of atomically thin Molybdenum Disulfide (MoS_2_) possess fascinating and unprecedented physico-chemical properties^1^. Along with attractive values of carrier mobilities, the possibility of achieving a current saturation in these materials indicate their potential use as thin film transistors and light emitting diodes^2^. Monolayer MoS_2_ is a direct band gap semiconductor (~1.8 eV) in contrast to its bulk counterpart (n-type semiconductor with an indirect band gap of ~1.3 ev), and therefore can be viewed as a valuable candidate for fabrication of components in electronic or optoelectronic devices^3^,^4^,^5^,^6^,^7^. Kim *et al.*^3^ have shown that by properly choosing the dielectric substrate, it is possible to attain high room temperature mobilities as well as very low sub threshold swing in few layer MoS_2_ field effect transistors. In addition, single and few layers of MoS_2_ exhibit impressive optical properties related to tunable photo detection^5^,^6^. These findings have resulted in the search for innovative methods related to the synthesis of MoS_2_ flakes of controlled thickness^8^,^9^,^10^,^11^,^12^,^13^,^14^,^15^. Methods such as laser thinning^11^, Chemical vapor deposition (CVD)^5^,^16^,^17^ and liquid phase exfoliation^8^ are now routinely used to obtain atom-thick layers of MoS_2_.

Among these techniques, liquid phase exfoliation such as the one reported by Coleman *et al.*^8^ is considered to be the most suitable method for achieving large scale and low cost synthesis of few layer MoS_2_. Since these materials are directly exfoliated in suspensions, they can be used as inks and can be used to cast thin films on a variety of substrates without any additional processing or transferring. In this letter we demonstrate that thin films of such liquid phase exfoliated MoS_2_ (see Methods for exfoliation details) can be used as photo-detectors with ultrafast photo-carrier generation and recombination times. These detectors can offer responsivity values of ~10^−4^ AW^−1^, higher than the responsivity values reported for CVD grown WS_2_ flakes^16^,^17^. Optical pump and Terahertz probe (OPTP) measurements indicate that the decay in the conductivity of the photo carriers closely follows a bi-exponential time dependence with a fast decay component τ_1_ ~ 5 ps and a slower component τ_2 _~ 110 ps. The slower decay component is a manifestation of electron-hole recombination in trap states (recombination centers) present between the valence band and the conduction band. We predict that these trap states are continuously distributed throughout the mobility gap of the photo-detector device. This prediction is made on the basis of photocurrent (I_ph_) measurements performed at low light power levels (20 μW < P < 570 μW, λ = 633 nm), which show that I_ph_ increases as a fractional power of light intensity over a broad range of temperature, a signature of continuous distribution of trap states^18^. In order to understand the role of these trap states in controlling the photocurrent behavior, temperature dependence of I_ph_ at a low light power of ~570 μW (λ = 633 nm) was measured. We observed that I_ph_ increased with decreasing temperature (from 330 K to 280 K) and reaches a maximum value at T = T_m_ ~  80 K. Thereafter I_ph_ decreases with further decrease in temperature, becoming greater than the dark current at ~120 K, and finally becomes temperature independent below 50 K. Such behavior is explained on the basis of the availability of trap states (acting as recombination centers) with the variation of temperature, and aligns well with established models of photo-carrier recombination processes in disordered semiconductors.

In Fig. 1 spectroscopy and microscopy characterization of the exfoliated MoS_2_ flakes are presented . A few droplets of the exfoliated MoS_2_ dispersion (Fig. 1a) were deposited and dried on SiO_2_ and analyzed by Raman spectroscopy. Raman measurements using the 514 nm laser excitation line (Renishaw Raman Spectrometer; Fig. 1b) were collected from several positions of the deposited film. A detailed analysis performed on the collected Raman measurements from the film and comparing it with on the available literature indicates that the exfoliated MoS_2_ flakes contain more than 4 layers (see supplementary information).

In Fig. 1c, we show the Tauc analysis of the UV-Vis spectra obtained from the MoS_2_ dispersions (details are provided in the supplementary section), which indicate that the dispersions possess an optical gap of ~1.74 eV. High Resolution Transmission Electron Microscopy (HRTEM) images of typical flakes are depicted in Fig. 1d–h. HRTEM images of the edges of some of these flakes (Fig. 1g,h) reveal the presence of multiple layers. These flakes were deposited on inter-digitated platinum electrodes (with a separation of 5 μm between two electrodes-see supplementary information for details of electrodes used.) on borosilicate glass substrates in order to form a thin film photo-detector. The MoS_2_ thin films deposited on the electrodes were then annealed at ~150 ^°^C for 2 hours (in an argon flow of 500 sccm) in order to improve the contact resistance between the MoS_2_ flakes and the electrodes. Finally, the electrodes were mounted inside a close cycle Helium cryostat (Janis Model # SHI- 4-1) with optical windows and pumped to high vacuum (~10^−5^ Torr) before performing the photoconduction measurements.

Room temperature current vs. voltage (IV) measurements without and with laser illumination was performed (using a Keithley Source meter 2400 Series) in order to investigate the nature of photoconduction in the device. A Coherent Cube solid-state laser system with a wavelength λ = 658 nm (E = 1.88 eV), 60 mW maximum power output and with a circular spot size of 3 mm diameter was used to illuminate the samples. The results of room temperature photoresponse investigation on these films are presented in Fig. 2a. The linear IV response with *light off* and *light on* under forward and reverse bias conditions and the absence of any zero-bias photocurrent in our devices (see supplementary information) indicate little or no photovoltaic contribution due to barrier effects occurring at the contacts, and thus the photoconduction is attributed to the photo carrier generation within the bulk portion of the MoS_2_ film. In order to investigate the photo switching behavior of the device at different light intensities, a 2 V DC bias was applied across the two terminals of the device and the corresponding current passing through the device without laser illumination (dark current; I_dark_) and with laser illumination (current under illumination; I_ill_) was recorded. Laser illumination of the device was switched ON and OFF in roughly 1000 ms intervals with varying laser output power from 0.67 mW to 60 mW. This data is presented in Fig. 2b. Fast photo switching of the detector on the order of ≤100 ms was observed, and it is of the similar order as previously reported in the case of single-layer MoS_2_ phototransistors^5^. In order to determine the intrinsic photo-carrier recombination times, instantaneous transient THz conductivity in exfoliated MoS_2_ films was measured using OPTP, described in detail later.

Along with the fast photo switching of these thin film photodetectors, a non-linear dependence of light intensity on I_ill_ was observed. We found that the photocurrent (I_ph_ = I_ill_ – I_dark_) follows a power dependence following I_ph_ ~ P^γ^, where P is laser intensity with γ = 0.66 (Fig. 2c). This behavior is different for simple crystals which typically possess γ = 0.5, corresponding to bimolecular processes, or γ = 1, corresponding to monomolecular processes. The monomolecular and bimolecular recombination processes are the two extreme cases observed in simple crystalline semiconducting systems with few trap states. We would like to note that several processes could lead to nonlinear power dependence of photocurrent. For example, photo-gating artifacts, thermoelectric effects, trapping by mid-gap states etc. comprise such processes. Our detailed investigations (described later in the manuscript) related to the variation of the power exponent with temperature as well as the comparative behavior of dependence of dark current and photocurrent with temperature strongly suggests that the photoconduction phenomenon in our films are similar to those obtained in the case of disordered semiconductor. Nevertheless, in order to verify that photo gating effects are not contributing to any photocurrent in our devices we have measured photocurrent by impinging the laser spot on the substrate in close proximity of the electrodes covered with MoS_2_ film. The device was then slowly moved under the laser illumination, such that the laser spot traversed from one end of the bare substrate over the device to the other end of the bare substrate (please refer to supplementary figure S8). The current response from the device was measured throughout this process. We found that an increase in current was only observed when the laser spot was on the sample, indicating that absorption of light in the substrate or by charges that reside near the device does not cause any changes in the photocurrent. Further, we have also noted that the photocurrent decay are fast, without any prolonged photocurrent decay tail (which arises if significant thermal effects are present), which suggests that contributions from thermoelectric effects are perhaps negligible. These observations leads us to believe that the fractional power dependence of photocurrent with laser intensity seen is indicative of the presence of a large number of trap states within these films^18^ (as explained in detail later).

We have also evaluated the responsitivity and external quantum efficiency of these samples at different laser powers. Responsivity is defined as R =I_ph_/P_light_ (where P_light_ is the power of the incident light)^5^, and is an important performance parameter of any photo detector. Figure 2d shows the responsivity of the exfoliated MoS_2_ films measured at different laser power with an applied bias of 2 V. The responsivity at the lowest power (0.67 mW) was found to be ~50 μAW^−1^ with a corresponding external efficiency of ~10^−2^%. This low external quantum efficiency is due to the highly disordered nature of the film. By increasing the applied bias to 20 V and the laser excitation power to 60 mW, a responsivity of R ~ 0.1 mAW^−1^ can be obtained (supplementary information). However this is significantly lower than the reported values for single- and few-layered MoS_2_ FET devices^5^,^6^, but is considerably higher than multilayer WS_2_ based devices^16^. Further, these R values are also similar to the values obtained on exfoliated MoS_2_/ITO junction photodetectors^17^.

As mentioned earlier, the fractional light intensity dependence of the photocurrent is due to the presence of trap states in the exfoliated MoS_2_ film. We provide further evidence of the presence of these trap states by measuring the photo carrier decay dynamics using OPTP. For this purpose, a ~70 femtosecond, 400 nm, 1kHz pump pulse with a fluence of 0.8 mJ/cm^2 was used to photo excite electron and hole pairs in ~2.5 mm diameter spot in these films. A sub-picosecond THz probe pulse, derived from the same laser system, was generated using optical rectification in a ZnTe nonlinear crystal (see supplementary information for schematic of the measurement setup). The instantaneous transient THz conductivity of the photo-carriers (△σ) was measured by recording the change in transmission of the peak of the THz pulse (△T/T) as a function of pump probe delay. In general, for thin films, △σ ~ −△T/T^19^. Thus, the generation of mobile photo-carriers results in a decrease in transmission of the THz peak, i.e. a negative OPTP signal, which eventually recovers to zero as the carriers lose mobility due to trapping or recombination.

In our measurements, carriers were measured as a function of the pump probe delay. We found that the decay in conductivity of the photo-carriers follows a bi-exponential time dependence with a fast decay component τ_1_ ~ 5 ps, and a slower decay component τ_2_ ~ 110 ps, indicating the presence of multiple relaxation processes (Fig. 2e). The time scales observed in our samples are similar to those found in the case of photo excited carrier relaxation times of mechanically cleaved single and few layers of MoS_2_ crystals, where the fast initial decay time τ_1_ (~few ps) is a signature of photo carriers being trapped by trap states^20^ and the longer decay time τ_2_ (~few hundreds of ps) is manifestation of photo carrier recombination. The dynamics of the photo carriers, which ultimately determines the performance of these materials in developing optical devices with ultrafast response times, depends on the distribution and nature of these trap states in the forbidden zone. The presence of sub-gap trap states are often found in thin films of exfoliated 2-D materials, for example reduced Graphene Oxide (rGO) films where the transport mechanism is mediated through a Mott-type variable-range hopping across sub-gap energy values^21^. Since few-layered MoS_2_ is a material of high importance in the next-generation of optoelectronic devices, understanding the nature of these sub-gap states is of vital importance from fundamental as well as applied perspectives. An understanding of the distribution of trap states as well as the availability of trap states in the photo conduction process is therefore necessary, and this can be determined by systematically monitoring the temperature dependence of the power exponent γ^22^ and by studying the temperature dependence of I_ph_ at fixed low light intensities^23^.

In order to understand the distribution of the trap states in the MoS_2_ photo detectors, we have performed measurements of I_ph_ at very low light intensities and at different temperatures. For this purpose a Uniphase Helium Neon Gas Laser (λ= 633 nm, E = 1.95 eV) with a maximum power output of 1mw was used. The results are depicted in Fig. 3. First, we confirmed the photo response of the device by applying a DC bias of 500 mV under a laser illumination power of 575 μW at various temperatures. The photo response under such conditions is presented in Fig. 3a. The dependence of I_ph_ on the laser power P (20 μW < P < 575 μW) again showed fractional power dependence of the form I_ph_ ~ P^γ^ (Fig. 3b) with 0.5 < γ <1.0 over a broad range of temperatures. A similar dependence of photocurrent is typically observed in a wide variety of disordered semiconductors and can be explained by the existence of a large number of trap states between the valence and conduction bands^18^,^23^. The disordered nature of the film therefore should give rise to a large number of localized trap states in the mobility gap. These trap states, which lie between the conduction band and the steady state Fermi level for electrons ((SSFL)n), do not take part in the recombination process since electrons falling into these states are rapidly re-excited thermally into the conduction band. A similar reasoning is also applied to trap states which lie between the valence band and steady state Fermi level for holes ((SSFL)p). However, the states lying between the SSFLs are responsible for recombination of electrons or holes. These states are designated as recombination centers; the free charge carriers falling into these states will generally recombine and will not contribute to the conduction. Under these assumptions, once the excitation is increased (for example by increasing the laser intensity), the number of free carriers increase and the two steady-state Fermi levels move apart toward their respective band edges, increasing the number of recombination centers (a phenomenon also known as electronic doping)^24^. The increase in the number of recombination centers reduces the lifetimes of the free carriers, thereby causing a sub-linear dependence of photocurrent on the incident light intensity values, which is what has been observed in our measurements. A simple schematic showing this process is represented in Fig. 3d.

Furthermore, the nature of the distribution of the trap states could be determined from the temperature dependence of γ. In particular, Haynes *et al.* have shown^25^ that it is possible to predict the distribution of the localized states by monitoring the value and temperature dependence of γ at low laser powers. For example, it is noted that a temperature dependence of the form γ = T_c_/(T + T_c_), where T_c_ is a characteristic temperature, indicates that the trap states are perhaps distributed exponentially. Our measurements indicate values of γ ranging between 0.5 and 1.0, over a wide range of temperature (30 K < T <220 K), and follow a similar temperature dependence when T_c_ ≅ 300 K (see Fig. 3c). In general the dependence of γ on temperature, as seen in our measurement, is strongly associated with a continuous distribution of trap states of the form ~exp(–ΔE/KT) below the conduction band, where ΔE is the energy separation measured from the bottom of the conduction band^18^. Therefore, we can confirm that in few-layered MoS_2_ thin film photo detectors, the trap states are continuously distributed.

In order to understand how the trap states control the photocurrent of these devices over a wide range of temperature, we have measured the dependence of I_ph_ with temperature (25 K < T <320 K) by applying a DC bias of 500 mV, under a constant illumination (λ = 633 nm, E = 1.95 eV) at 575 μW. The data is presented in Fig. 4, where ln(I_ph_) is plotted as a function of 1/T and it is compared with the value of I_dark_. Several interesting features of I_ph_ can be seen from this measurement. Firstly, depending on the measurement temperatures, two distinct regions for I_ph_ can be identified: Region I (T <120 K; where I_ph_> I_dark_) and Region II (T >120 K; where I_ph_< I_dark_). Secondly, at low temperatures (T <50 K), I_ph_ is constant and thirdly, in Region II (T >120 K), I_ph_ shows a maxima at T = T_max_ ~ 280 K.

These behaviors can be understood by considering the position of the SSFLs as described under the model proposed by Rose^18^. At low temperatures (Region I, T <120 K) the SSFL’s are closer to each other (away from their respective band edges). Under these conditions, in our system, the number of photo-generated carriers (N_ph_) is significantly greater than the number of trap states acting as recombination centers (N_rc_). This leads to the condition where I_ph_ > I_dark_. At sufficiently low temperatures (T ≤ 50 K), a constant I_ph_ is also observed, indicating that a constant photo carrier density is reached in these photo detectors. As the temperature increases, the two SSFL’s move apart toward their respective band edges, thus increasing the number of recombination centers. Under these conditions, N_rc_ >> N_ph_ and thus higher recombination of the generated photo carriers occur, resulting in I_ph_ becoming < I_dark_ (as seen in region II (T ≥ 120 K)). Further in this region, I_ph_ shows a maxima at T = T_max _~ 280 K (Inset Fig. 4). In the past, it has been shown for several photoactive disordered semiconductors that although the photo current on both sides of T_max_ is carried through extended states, as the temperature increases (for T >T_max_) the number of thermally generated carriers also increase^26^. This in turn increases the dark current and results in the decrease of I_ph_ once T_max_ is reached.

In conclusion we have shown that films of few-layered liquid phase exfoliated MoS_2_ are suitable candidates for fabricating fast response photo detectors. Detailed photocurrent measurements as a function of temperature show that these films contain extended trap states, which mainly arise due to the disorder caused by the random orientation of the stacked layers of the exfoliated flakes. This results in a faster recombination of the charge carriers, but also reduces the responsivity of these materials when compared to several recently studied mechanically exfoliated and Chemical Vapor Transport grown 2D layered chalcogenides^27^,^28^,^29^,^30^,^31^,^32^,^33^,^34^,^35^. However, we have also shown that under certain conditions, responsivities higher than few layer CVD grown WS_2_ photo detectors is possible, but overall improvements in responsivity by further tuning the exfoliation process could be reached in the future. A table (supplementary information) detailing the responsivity of several single and few layer Chalcogenides is also presented for comparison. One important aspect of these findings is directly related to the fabrication of low-cost photo-detectors or electrode materials for low-loss solar power conversion. In order to optimize the performances of such devices, a clear understanding of the photo carrier dynamic and the role of sub gap states present in these materials is essential. We believe that the experimental evidence related to the inherent presence of sub gap states in liquid-phase exfoliated 2D MoS_2_ flakes sheds light on several aspects of their photo response behavior. The results presented in this manuscript will not only open up similar investigations, but also will motivate the development of photoactive nano-materials and composites for several opto-electronic applications.

## Methods

The synthesis of MoS_2_ dispersions was performed via a liquid phase exfoliation technique described by Coleman *et al.*^8^. Commercial MoS_2_ powder (Sigma Aldrich, <2 μm) was added to isopropanol alcohol (IPA) at a concentration ratio of 10 mg/mL. The mixture was then treated with ultrasound from a Fisher Scientific Sonic Dismembrator 500 horn tip sonicator operating between 20% to 40% amplitude. The dispersions were treated with ultrasound for 4 hours continuously, using an ice water bath to maintain the temperature. After the ultrasound treatment, the dispersions were centrifuged at 1500 rpm for 45 minutes and were then decanted. The dispersion thus obtained was used to fabricate the photo detector device. This was achieved by depositing a small droplet of the dispersion containing the exfoliated MoS_2_ on prefabricated inter-digitated electrode assembly with inter-digitated finger separation of 5 μm (ALS Japan). The devices were then annealed at 150 ^°^C under an Argon atmosphere for 2 hours. Annealing leads to the reduction in the amount of chemical impurities, surface ad-atoms etc. present in the samples due to the exfoliation process thereby making the metal sample interface less resistive. A schematic of the whole process is shown in the supplementary information. After the annealing the device was mounted on a closed cycle Helium Cryostat (Janis Model SHI) and was pumped down (pressures typically below 10^−5^ Torr) overnight before performing optical measurements.

## Additional Information

**How to cite this article**: Ghosh, S. *et al.* Ultrafast Intrinsic Photoresponse and Direct Evidence of Sub-gap States in Liquid Phase Exfoliated MoS_2_ Thin Films. *Sci. Rep.* **5**, 11272; doi: 10.1038/srep11272 (2015).

## Supplementary Material

Supplementary Information

## Figures and Tables

**Figure 1:**
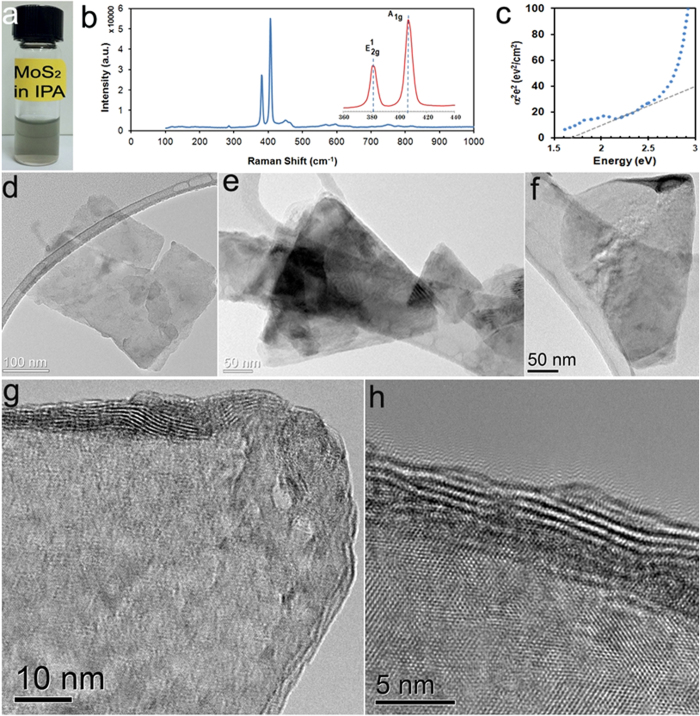
Synthesis and characterization of liquid phase exfoliated MoS_2_. (**a**) Digital image of exfoliated MoS_2_ dispersion in IPA. (**b**) Raman spectra measured from the samples. (**c**) Tauc plot extracted from the UV-Vis spectrum of the exfoliated samples. (**d**) (**e**) & (**f**) TEM images of typical flakes. (**g)** & (**h)** shows the HRTEM images of exfoliated flakes showing few layers as well as a highly crystalline structure.

**Figure 2:**
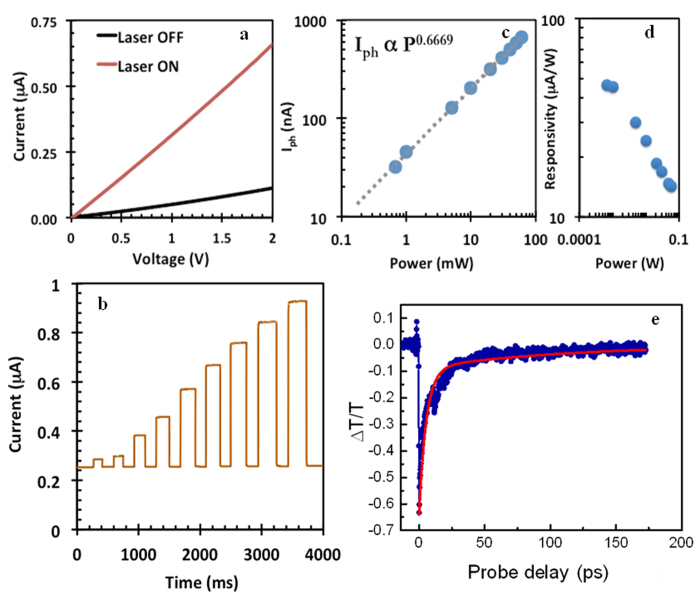
Electrical and optical measurements on liquid phase exfoliated MoS_2_ films. (**a**) IV measurement on MoS_2_ device, with light off and light on (**b**) photo switching at ~283 K for (from left to right) 0.67, 1, 5, 10, 20, 30, 40, 50 and 60 laser power (in mW). (**c**) Variation of I_ph_ with laser intensity (at 60 mW). (**d**) Detector responsitvity as a function of laser power. (**e**) Transient Differential THz Transmission proportional to photocarrier conductivity (△T/T ~ −△σ) measurement as a function of probe delay time, showing a bi-exponential decay in photoconductivity (fit solid line).

**Figure 3:**
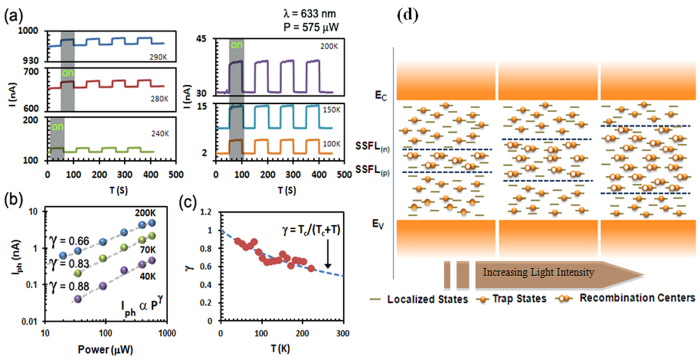
Temperature dependence of optical properties of liquid phase exfoliated MoS_2_ films. (**a**) The photo response of the device measured (applied voltage 1 V, λ = 633 nm, P = 575 μW) at several temperatures is presented. (**b**) Dependence of photocurrent with respect to low light intensity on the same device (applied voltage 0.5 V, λ = 633 nm) (**c**) Temperature dependence of power exponent γ under low light condition. (**d**) Schematic representation of the movement of Steady State Fermi Levels with increases light intensity.

**Figure 4:**
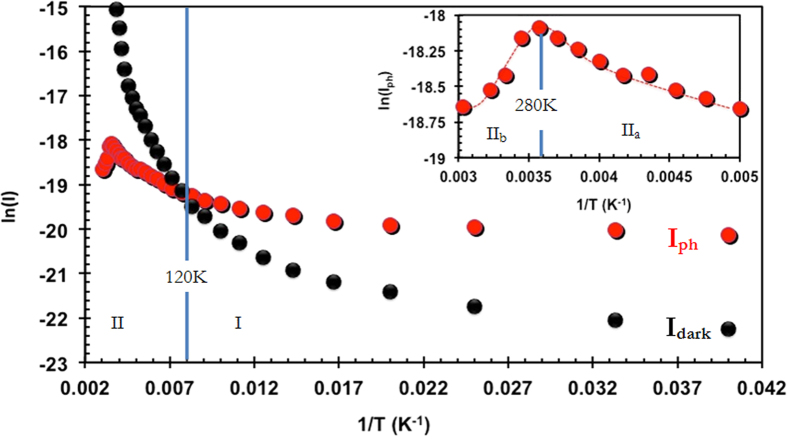
Temperature dependence of photocurrent. Temperature dependence of photocurrent and dark current as seen in thin film MoS_2_ photo-detectors. A maximum of photocurrent at high temperatures (inset) as well as higher value of photocurrent compared to dark current at lower temperatures.
