# Supplementary material for: Ultrafast Intrinsic Photoresponse and Direct Evidence of Sub-gap States in Liquid Phase Exfoliated MoS2Thin Films
Source: Sci Rep. 2015 Jul 15;5:11272. doi: 10.1038/srep11272 (PMC4502394; doi:10.1038/srep11272)
Supplement: Supplementary Information [file srep11272-s1.docx]

**Supplementary Information**

Ultrafast Intrinsic Photoresponse and Direct Evidence of Sub-gap States in Liquid Phase Exfoliated MoS_2_Thin Films

Sujoy Ghosh^1^, Andrew Winchester^1^, Baleeswaraiah Muchharla^1^, Milinda Wasala^1^, Siming Feng^2^, Ana Laura Elias^2^, M. Bala Murli Krishna^3^, Takaaki Harada^3^, Catherine Chin^3^, Keshav Dani^3^, Swastik Kar^4^, Mauricio Terrones^2,5^, and Saikat Talapatra^1,3^

^1^Department of Physics, Southern Illinois University Carbondale, Carbondale-IL 62901.

^2^Department of Physics and Center for 2-Dimensional and Layered Materials, The Pennsylvania State University, University Park, PA 16802

^3^Femtosecond Spectroscopy Unit, Okinawa Inst. of Science & Technology, Graduate University, Onna-son, Okinawa, Japan 904 -0495

^4^Department of Physics and and George J. Kostas Research Institute, Northeastern University, Boston, USA

^5^Department of Chemistry and Department of Materials Science and Engineering, The Pennsylvania State University, University Park, PA 16802

**Exfoliation Method and Photo detector device fabrication:** The following figure shows the schematic of material synthesis and device fabrication.

**Figure S1**. Method of liquid phase exfoliation and procedure followed to make a simple photo detector device.


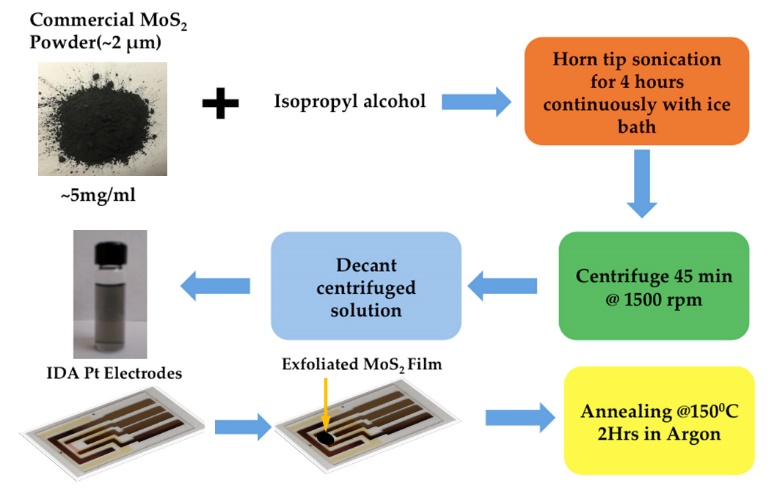


**Ultraviolet-visible (UV-VIS) spectroscopy:** UV-Vis spectroscopy of the dispersions was carried out with a Hach DR4000U Spectrophotometer. Absorbance spectra were taken from 190nm to 800nm. The absorption data is presented in figure S2. The direct band gap of the exfoliated material was estimated by means of a Tauc plot. In order to empirically determine the band gap energy E_g_ in the Tauc equation αE = A(E-E_g_)^r^, the quantity (αE)^1/r^ was varied against E, where α is the absorption coefficient, E is the photon energy, A is a constant and r is a constant depending on the dimensionality and type of transition. For direct transitions the value of r is ½. Fitting the linear absorption region with Tauc equation gives a good estimate of the optical band gap of the dispersion [S2].


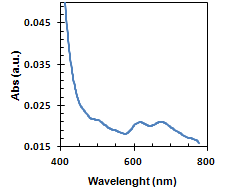


**Figure S2**. UV-Vis absorption spectra of exfoliated MoS_2_ solution.

**Raman Spectroscopy**: Raman measurements were performed using a Renishaw inVia Raman spectrometer with a 514.5 nm excitation wavelength. Extended scans on several exfoliated MoS_2_ flakes were performed. A representative Raman spectrum is depicted in figure S2, exhibiting the out of plane A1g mode (~407 cm^-1^) and the in plane E^1^_2g_ mode (~383 cm^-1^), which are the most common Raman signatures for MoS_2_. The frequency difference (Δω) between the A_1g_ and the E^1^_2g_ modes (a good indicator of number of layers present in MoS_2_), obtained from these scans was analyzed. This difference was found to be 24.4 cm^-1^<Δω < 25.5 cm^-1^, indicating that our exfoliated MoS_2_ samples perhaps contain more than 4 layers [S3]. The values of 24.4cm^-1^ and 25.5cm^-1^, for a wavelength of 514.5 nm correspond to the frequency difference of 4 layers and bulk MoS_2_, respectively. Several Δω data points obtained from the Raman measurements are presented in the inset of figure S3.

**Figure S3**. Raman spectra showing the E^1^_2g_ (~383 cm^-1^) and the A_1g_ (~407 cm^-1^) modes of the exfoliated MoS_2_ flakes. (Inset) The frequency difference values of these two modes obtained from Raman measurement performed at several different locations is plotted along with the frequency difference values of 4 layers as well as bulk MoS_2_ is shown.


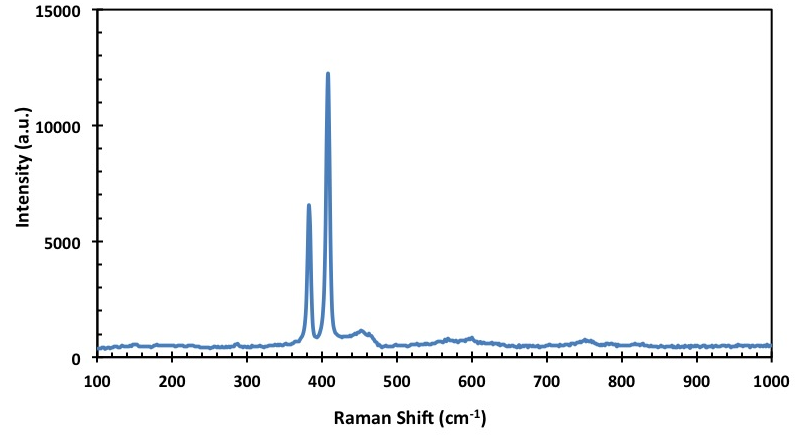

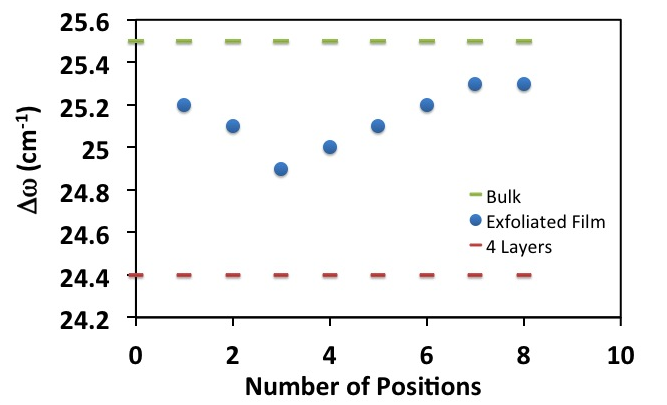


**High-resolution transmission electron microscopy (HRTEM):** For HRTEM imaging the sample was dropped on to lacey carbon copper TEM grid. TEM images and electron diffraction patterns were obtained using a JEOL 2010F with an accelerating voltage of 200kV, field-emission source, ultra high-resolution pole piece (Cs=0.5mm), and 1.9Å Scherzer limit. Acquisition times for TEM images were 1s per frame.

**Optical Pump and THz Probe (OPTP) measurements:** For the transient photoconductivity measurement, a Femto second pulse ~70fs, 400nm-pump, 1 kHz pump pulse with a power of 14mW photo excited electron and hole pairs in the films. A sub-picosecond THz probe pulse, derived from the same laser system, was generated using optical rectification in a ZnTe nonlinear crystal. A detailed schematic view of the optical arrangement of the OPTP system is shown in figure S4.

**Figure S4**. Schematics of the OPTP setup used for transient photoconductivity measurements.


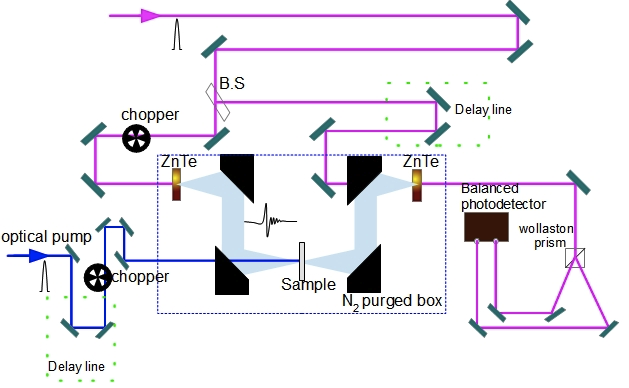


To calculate ΔT/T as plotted on the Y-axis of figure 2e, we first measure ΔT –the pump induced change in the transmission of the THz signal at the peak, by chopping the pump and recording the differential transmission using a lock-in amplifier. The total transmission T is simply given by the peak value of the THz time domain signal as shown in figure S5. For the THz measurements, the sample was prepared by drop casting MoS_2_ solution on a single crystal Z-cut Quartz substrate (1cm x 1cm).


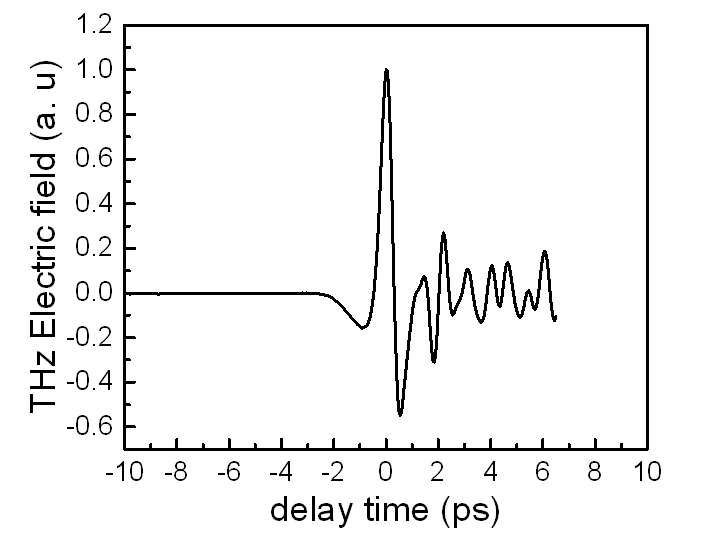


**Figure S5:** THz Time-Domain Electric field amplitude through MoS_2_ sample and single-crystal z-cut quartz substrate.

**Electrical transport measurements:**


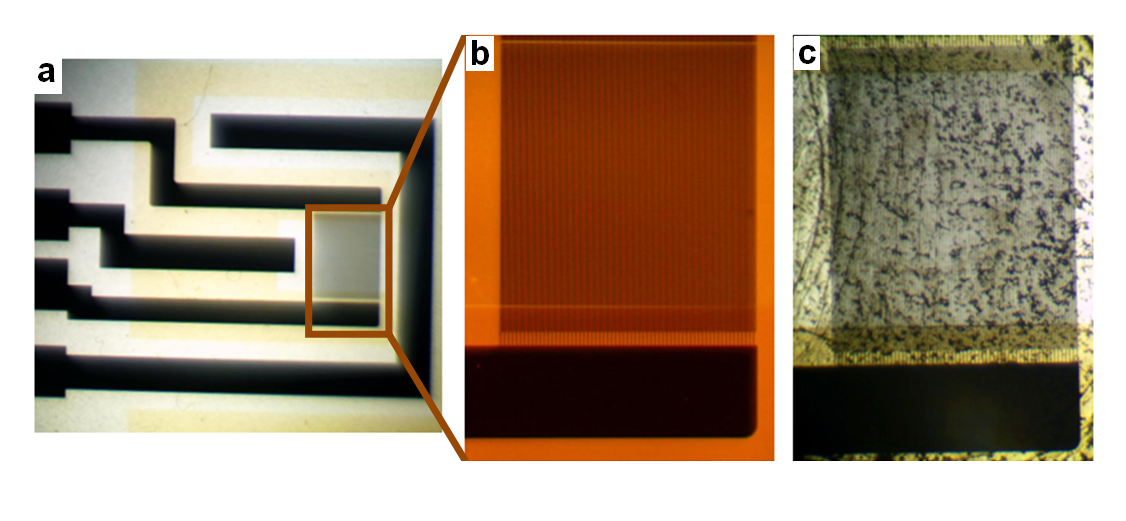
**Interdigitated Electrode:** The electrical and optical transport measurements were performed on liquid exfoliated sample films deposited on interdigitated electrodes. The interdigitated platinum electrodes employed for this purpose was purchased from CHI instruments (CHI#010380) made by ALS Co., Ltd. The electrode consists of an array of 65 pairs of inter-digitated line patterns with a separation of 5µm. Each line is 10µm wide and 2mm long. The whole pattern is fabricated on a glass substrate as shown in the following figure. Further detail of these electrodes can be found on <http://www.als-japan.com>. In figure S6 we have shown the interdigitated electrode pattern before and after drop casting the MoS_2_ flakes on it.

**Figure S6**. (a), (b) Interdigitated electrode without sample (c) with samples.

For measuring the IV responses, the devices were mounted on a cryostat and pumped over night under a vacuum of ~ 10^-5^ Torr. IV measurements were performed using a Kiethley 2400 series source meter. IV response with light off and light on under forward and reverse bias conditions were obtained using a laser line of λ = 658 nm at 60mW power (figure S7). The linearity of IV curves without illumination and with illumination is mentioned is extremely important. This shows that within the applied voltage regime, under which the measurements are performed, the contacts are able to replenish the photo-carriers when they are drawn out from the material under an applied electric field. Under such conditions we can assume that the contacts are “behaving” as “ohmic” rather than blocking or injecting. If the IV showed a less than linear variation in the current with applied voltage (and current saturates eventually) or the current increases rapidly (space charge limited) with applied voltage then clearly the contacts are behaving as “blocking contacts” or “injecting contacts” respectively. Since, the voltage regime with in which the measurements were performed we have not seen any non-linearity in the IV data. Thus, we strongly believe that the contact effects are negligible in our measurements." The linear IV response as well as absence of any zero-bias photocurrent in our device indicates little or no photovoltaic contribution due to barrier effects at the contacts. This manifests that the photoconduction is attributed due to the photo carrier generation within the bulk portion of the MoS_2_ film. Further, in order to verify that photo gating effects are not contributing to any photocurrent in one of our devices, we have measured photocurrent by impinging the laser spot on the substrate in close proximity of the electrodes covered with MoS_2_ film. The current response from the device was measured throughout this process. We found that an increase in current was only observed (figure S8) when the laser spot was on the sample, indicating that absorption of light in the substrate, or by charges that resides near the devices does not cause any changes in the photocurrent.


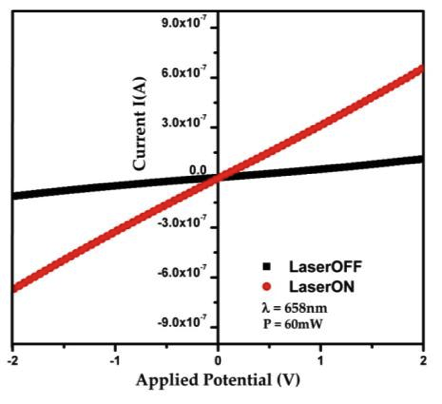


**Figure S7**. IV response of MoS_2_ without & with light is presented.

We have also provided some of the data presented in figure 2 and 3 in log-log scale in linear scale as shown in figure S9.


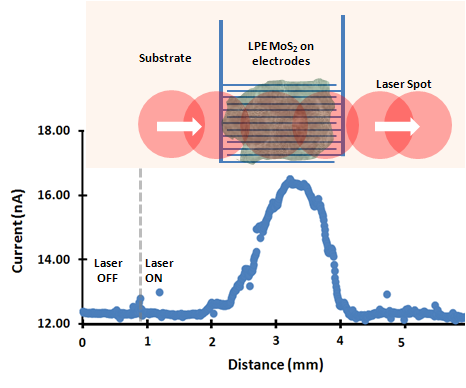


**Figure S8:** Photoresponse profile of a typical device under laser spot translation over the active area and the bare substrate.


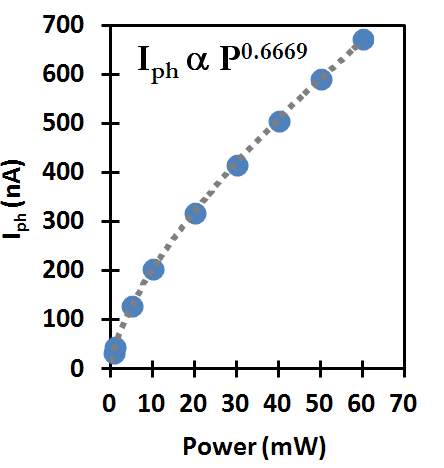

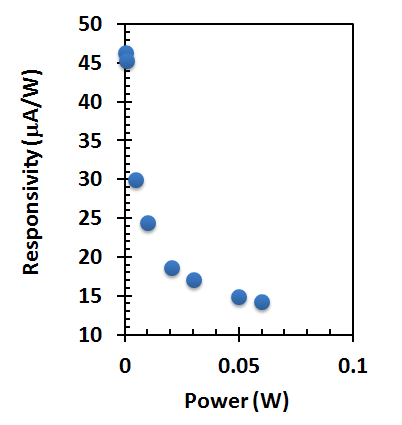

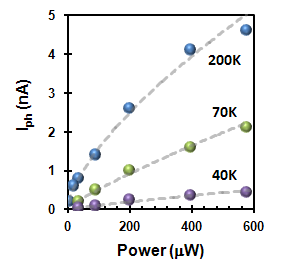


**Figure S9**. Photocurrent variation is shown in linear scale for data shown in the main manuscript for (a) figure 2 (c), (b) figure 2 (d) and (c) figure 3(b).

**(a)**

**(b)**

**(c)**

**Responsivity:** Responsivity, defined as R = I_ph_/P_inc_, was measured using a λ = 658 nm at 60mW power. The variation of R as a function of applied across the device was measure. It was observed that R ~ 0.14mAW^-1^ can be obtained at an applied bias of 20V, similar to responsivities seen in exfoliated MoS_2_ films [S4] on ITO (in junction solar cell geometry). The variation of R with applied bias is also shown in figure S10. We have measured three different devices and their response was found to be similar. This indicates the repeatability of the data presented.


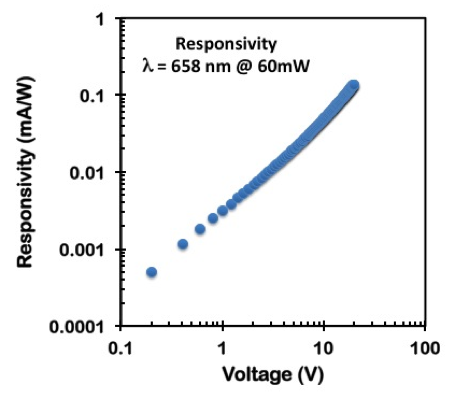


**Figure S10**. Variation of responsivity as a function of applied voltage across the device is shown.

| **Material** | **Device Architecture** | **Responsivity** | **Wavelength** | **Reference** |
| --- | --- | --- | --- | --- |
| **Monolayer MoS_2_** | **3 terminal FET**  **V_DS_ = 1V,V_G_= 50V** | **8mAW^−1^** | **532nm** | **[5]** |
| **Monolayer MoS_2_** | **3 terminal FET**  **V_DS_ = 8V,V_G_= -70V** | **880A*W*^-1^** | **561nm** | **[28]** |
| **Monolayer GaS** | **2 terminal**  **V_DS_ = 2V** | **4.2AW^-1^** | **254nm** | **[29]** |
| **Exfoliated MoS_2_ Nano-platelets** | **2 terminal**  **V_DS_ = 15V** | **10^-4^AW^-1^** | **White light** | **[17]** |
| **Multilayer MoS_2_** | **3 terminal FET**  **V_DS_ = 1V,V_G_= -3V** | **110 mAW^−1^** | **633nm** | **[30]** |
| **Multilayer WS_2_** | **2 terminal**  **V_DS_ = 30V** | **92 μAW^-1^** | **458nm** | **[16]** |
| **Multilayer In_2_Se_3_** | **2 terminal**  **V_DS_ = 5V** | **395AW^-1^** | **300nm** | **[31]** |
| **Multilayer GaTe** | **2 terminal**  **V_DS_ = 5V** | **10^4^AW^-1^** | **532nm** | **[32]** |
| **Multilayer GaSe** | **2 terminal**  **V_DS_ = 5V** | **2.8AW^-1^** | **254nm** | **[33]** |
| **Multilayer GaSe** | **2 terminal**  **V_DS_ = 10V** | **17mAW^-1^** | **405nm** | **[34]** |
| **Multilayer InSe** | **2 terminal**  **V_DS_ = 3V** | **34.7mAW^-1^** | **532nm** | **[35]** |
| **Multilayer TiS_3_**  **nanoribbon** | **3 terminal FET**  **V_DS_ = 1V,V_G_= -40V** | **2910AW^-1^** | **640nm** | **[36]** |

REFERENCES

S1. Coleman, J. N. et al. *Science*. **2011**, 331, 568-571.

S2. Dholakia, D. A.; Solanki, G. K.; Patel, S. G.; Agarwal, M. K.; Bull. Mater. Sci. **2001,** 24, 291–296.

S3. Li, H.; Zhang, Q.; Chong R-Y, C; Kang T, B.; Teo H-T, E. ; Olivier, A.; Baillargeat, D.

*Adv. Funct. Mater.* **2012**, 22, 1385–1390.

S4. Cunningham, G.; Khan, U.; Backes, C.; Hanlon, D.; McCloskey, D.; Donegan, J. F.; Coleman, J. N. ***J. Mater. Chem. C*,** **2013**, **1,** 6899-6904.
